# Double primary malignant fibrous histiocytoma and squamous cell carcinoma of the larynx treated with laser laryngeal conservation surgery

**DOI:** 10.3332/ecancer.2016.636

**Published:** 2016-04-25

**Authors:** PD Karkos, S Dova, S Sotiriou, K Markou, I Kostopoulos

**Affiliations:** 1 Department of Otolaryngology-Head Neck Surgery, Ahepa University Hospital, Thessaloniki 546 21, Greece; 2 Department of Histopathology, Aristotle University of Thessaloniki, Thessaloniki 546 21, Greece

**Keywords:** fibrous histiocytoma, squamous cell carcinoma, larynx, laser

## Abstract

**Βackground:**

Synchronous multiple malignancies of the larynx are rare. We present a case here of synchronous primary laryngeal squamous cell carcinoma (SCC) and malignant fibrous histiocytoma (MFH) in a patient with hoarseness though with no history of exposure to radiation. Clinical, intraoperative, and histopathological findings in this patient are discussed.

**Methods:**

Wide laser excision of the left supraglottic lesion and laser cordectomy of the right true vocal cord were performed.

**Results:**

The patient presented with a recurrence of the ΜFH alone (with no recurrence of the SCC) two months after the first operation and was managed with an extended second look laser cordectomy. The patient is under regular follow-up and remained disease-free nine months from diagnosis.

**Conclusions:**

Our results show that early-stage simultaneous tumours of the larynx and particularly MFH and SCC can be treated efficiently with endoscopic laryngeal surgery alone. Close follow-up is of paramount importance because of the aggressive nature of MFH.

## Introduction

The first case of multiple primary tumours was reported in 1889 by Billroth [1]. Synchronous multiple primary malignancies of the larynx are very rare. Only very few simultaneous multiple malignancies of the larynx have been reported to date [2]. We report here a case of synchronous laryngeal SCC and primary MFH.

## Case report

**Figure 1.**
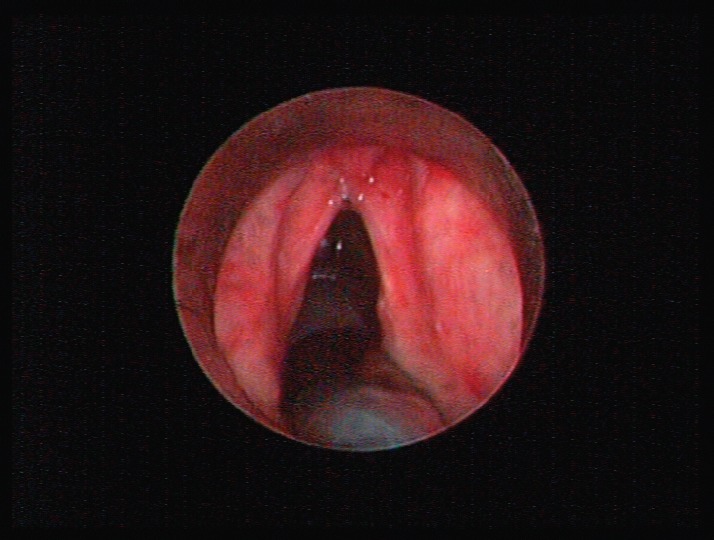
A mass emanating from the supraglottic part of larynx without erosion or haemorrhage and a lesion of the true right vocal cold presenting as leukoplakia.

**Figure 2.**
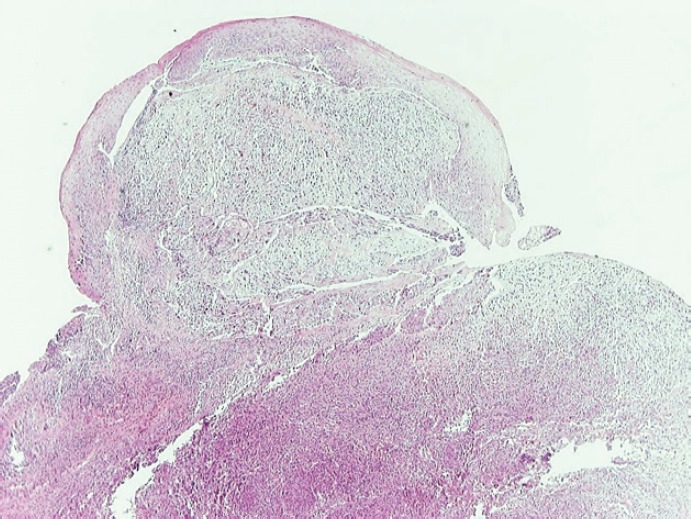
Low-power view of the mass shows two neoplastic populations (sarcomatoid and epithelial component) and the overlying dysplastic epithelium (H&E, X40).

**Figure 3.**
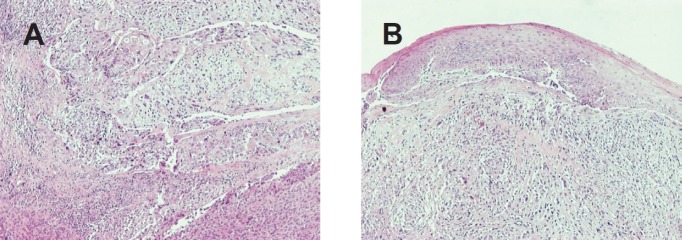
(A) Low-power view demonstrates the neoplastic populations to intermingle (H&E, X100). (B) Low-power view shows the sarcomatoid component and the overlying epithelium (H&E, X100).

A 69-year-old man with a history of smoking and alcohol consumption was referred to otolaryngology complaining of hoarseness for almost one year. Full head and neck examination was performed including flexible laryngoscopy revealing a mass emanating from the left supraglottic part of larynx without erosion or haemorrhage and a lesion of the true right vocal cold presenting as leukoplakia (Figure 1). Bilateral vocal cord mobility was normal. Computerised Tomography(CT) of the neck and chest were normal without any lymph nodes or distant metastases. Furthermore laboratory tests, chest x-ray, and abdominal ultrasonography were also normal. There was no previous history of radiotherapy to the neck. Microlaryngoscopy and biopsy were performed (Figure 1). Histopathological analysis demonstrated a double malignancy in the left supraglottic lesion, i.e. a moderately differentiated SCC and MFH. Sections stained with haematoxylin and eosin from the mass of the left supraglottic area showed a biphasic neoplasm composed of two populations in close relation, which covered at least focally by stratified squamous epithelium, with mild to moderate dysplasia (Figures 2 and 3)

The prominent neoplastic population consisted mainly of plump spindle cells arranged in short fascicles in a storiform pattern. A portion of these cells were arranged in a diffuse growth pattern. In these areas, the neoplastic cells had hyperchromatic, irregular nuclei with prominent one or more nucleoli. Giant, bizarre, or multinucleated cells were commonly along with typical and atypical mitotic figures. Aggregates composed predominantly of lymphocytes and plasma cells and strands of collagen were seen in the stroma. The immunophenotype (Table 1) and the morphological features of this neoplastic population were compatible with a pleomorphic undifferentiated sarcoma/malignant fibrous histiocytoma (PUS/MFH).

**Figure 4.**
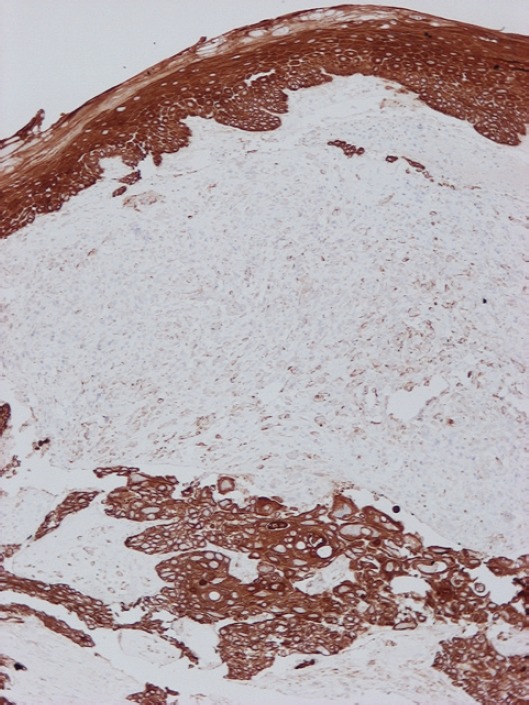
Diffuse immunoreactivity of squamous cell carcinoma and the overlying epithelium for cytokeratin AE1/AE3 and focal/weak immunoreactivity of the sarcomatoid component (Immunoperoxidase with haematoxylin counterstain, X100).

The second neoplastic population consisted of epithelial component and corresponded to a moderately differentiated SCC. The neoplastic cells were arranged in solid masses or cords and had eosinophilic or clear cytoplasm and big irregular nuclei with prominent nucleoli. Intercellular bridges were present. The immunophenotype of this population was typical to that of any SCC (Table 1). The cells of the two populations intermingled (Figure 4).

**Figure 5.**
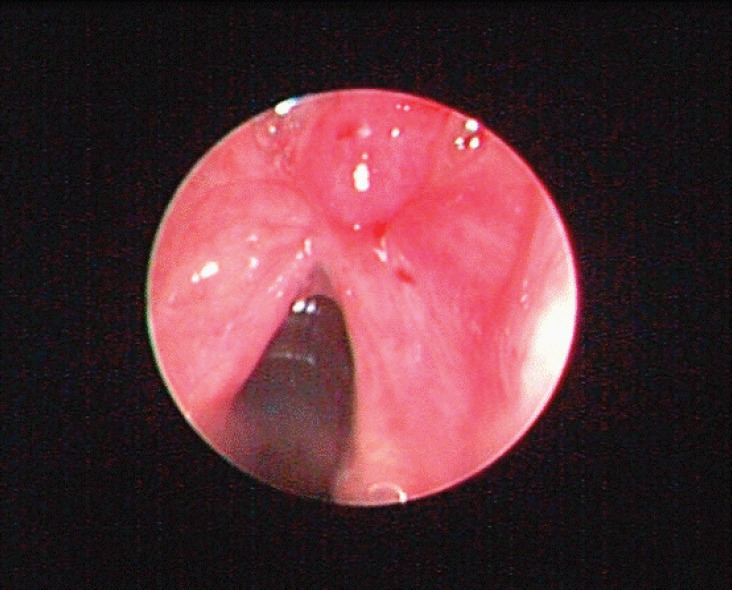
Small supraglottic mass just above the anterior commissure two months after initial treatment was excised with CO2 laser and proved to be a recurrence of histiocytoma. The patient remained disease-free nine months after the second look cordectomy.

A wide laser cordectomy of the left supraglottic mass and the right vocal cord with CO2 laser were performed. The results of the right true vocal cord biopsy were compatible with moderately differentiated SCC. During follow-up period, two months after the operation a very small granulomatous-type exophytic mass was found just above the anterior commissure (Figure 5). An extended second look laser cordectomy was performed and final histopathological analysis confirmed once again MFH. The patient is under regular follow-up and nine months later remained disease-free.

## Discussion

SCC represents over 95% of all laryngeal malignant tumours. MFH is a non-epithelial tumour of mesenchymal origin that is composed of a variety of cell types, such as fibroblasts, atypical giant cells, and histiocyte-like cells. MFH is usually localised in the limbs, the abdomen, and the retroperitoneum. Almost 3–10% of all MFH are localised in the head and neck region. The most frequent localisations are sinonasal cavities (30%), craniofacial bones (15%), and larynx (10–15%) [3, 4]. MFH represents approximately 2% of all malignancies in the larynx. The number of cases found in the literature is limited [5].

The first case of laryngeal MFH was described in 1972. Laryngeal MFH is more prevalent in men than in women with a ratio of 3:1, and it usually appears in adults of average age of 43 with a wide age range [4]. In men MFH is found typically in glottis, whereas in women in the subglottis [3, 4]. The majority of cases with laryngeal MFH are associated with radiation [6]. However, in our patient there was no previous exposure to radiation. This suggests that the aetiology of MFH needs further analysis. Prognostic factors of MFH are tumour size, gender, age, histological grade, metastasis, and vascular invasion [2, 7]. The most common symptoms of laryngeal MFH are hoarseness, difficulty in swallowing, and dyspnea. Symptomatology depends on the size and location of the lesion. MFH presents in the larynx as a polyp or a nodule. Radiological findings characteristic of MFH do not exist. Although diagnosis is difficult with conventional histopathology, immunohistochemistry helps to discriminate MFH among the tumours of mesenchymal origin. Histological diagnosis includes proliferation of neoplastic cells that resemble fibroblast and histiocytes with abnormal atypias and mitosis. These are organised in bundles. The most common type is the storiform one which produces a collagen matrix.

The immunohistochemical analysis in this case was crucial for the final diagnosis. In the case of the spindle/pleomorphic population the absence of any immunoreactivity for high molecular weight cytokeratin 34βΕ12 (which consist of cytokeratin 1, 5, 10, and 14) and cytokeratin 5/6, along with the at least focal positivity for markers of mesenchymal differentiation (vimentin, smooth muscle actin [SMA], desmin, sarcomeric actin, neurofilaments) excluded the diagnosis of a SCC. On the other hand, the absence of specific myogenic, vascular or neuromatous differentiation (only focal immunoreactivity for these markers) lead to the diagnosis of PUS/MFH.

Focal or weak immunoreactivity for mesenchymal markers such as vimentin [8, 9], SMA [10], CD10 [11, 12], CD99 [13], CD68 [14, 15], lysozyme [16, 17], fascin [18], and other intermediated filaments like desmin [10, 19, 20] and neurofilaments [9, 19] has already observed in cases of PUS/MFH. Additionally, cases of PUS/MFH positive for cytokeratin and EMA have been reported in the literature [9, 10, 19, 20, 21, 22, 23]. Positivity for protein p63 is extremely rare [24]. Although there are no previous reports for immunoreactivity for the antigen CD31 in PUS/MFH, however, CD31 positivity highlighting cutaneous histiocytomas and histiocytoma mimics [25] CD31 immunoreactivity. On the other hand, only sarcomatoid SCC shows positivity for markers such as vimentin, SMA, CD10, CD99, CD68, lysozyme, fascin, desmin, and neurofilaments [26, 27, 28, 29, 30].

In 1889, the first case of multiple primary tumours was reported. Primary multiple malignant tumours of the larynx are extremely rare. On reviewing the literature we only found seven cases, and to our knowledge our case is the second report of synchronous laryngeal SCC and MFH [2]. The supraglottis is a rare location. Surgery is the treatment of choice for MFH, and it depends on location and size of the tumour, as happens with SCC. Radiotherapy or chemotherapy is not recommended as it does not offer any benefits to surgery. Wide removal with free clear margins is desirable. MFH is a very aggressive tumour [31]. The most common metastases are in the lung, liver, and spleen [32]. Close and frequent follow-ups are obligatory so that recurrences can be detected early and treated efficiently [31, 33]. In our case the extended supraglottic laser cordectomy which was performed, ensured a complication-free postoperative course and at nine-month follow-up the patient remained disease-free. In general, survival for MFH is approximately 60% after five years and 40% after ten years.

## Conclusion

In conclusion synchronous multiple malignancies of the larynx are uncommon. We report here a case of a laryngeal SCC, moderately differentiated, with simultaneous primary MFH which was confirmed with histopathological and immunohistochemical examination. Our results show that early-stage of simultaneous tumours in the larynx and in particular the MFH and SCC can be removed via laser laryngeal conservation surgery. Close follow-up is mandatory in order to detect early recurrences or a distal metastasis, considering the fact that MFH is an aggressive tumour.

## Figures and Tables

**Table 1.** Results of immunohistochemical analysis.

Antibodies	Results	
	SCC	PUS/MFH
Cytokeratin AE1/AE3	+	--/+
Cytokeratin 5/6	+	-
Cytokeratin 34βΕ12	+	-
p63	+	--/+ (second biopsy)
EMA	-	-
Vimentin	-	+
CD31	-	--/+
CD34	-	-
Factor VIII	-	-
SMA	-	Rare positive cells
Desmin	-	Rare positive cells
Sarcomeric actin	-	Rare positive cells
Myoglobin	Not done	Not done
Neurofilaments	-	Rare positive cells
CD57	-	-
S-100	-	-
CD1a	-	-
CD68	-	--/+
Lysozyme	-	++/-
Fascin	-	+
CD10	-	++/-
BCL-2	-	--/+
ALK1	-	-
CD99	-	+
β-Catenin	-	-
Ki67/MIB1	25%	90%
